# Dealing With Loneliness Among LGBT Older Adults: Different Coping Strategies

**DOI:** 10.1093/geroni/igaa057.1667

**Published:** 2020-12-16

**Authors:** Natasha Peterson, Jeongeun Lee, Daniel Russell

**Affiliations:** 1 Iowa State University, Ames, Iowa, United States; 2 Iowa State University, Iowa City, Iowa, United States

## Abstract

Older lesbian, gay, bisexual, transgender (LGBT) adults may be at risk for high levels of loneliness—a risk factor for worse health behaviors—as a result of historical and social discrimination. Some LGBT older adults may have estranged relationships with family members or have toxic relationships, consequently leaving them without adequate social support. The 2018 Loneliness and Social Connections survey by the AARP Foundation consists of a national sample of non-institutionalized individuals 45 and older, including 2905 individuals who identify as heterosexual and 318 who identify as LGBT. The study indicated individuals had similar levels of loneliness regardless of sexual orientation. However, significant differences between heterosexuals and LGBT participants were found in their communication and time usage when they are lonely. For example, heterosexual individuals socialize with friends in person more than homosexuals (t=-2.393, p<.05), whereas LGBT older adults use technology more to socialize with friends (t=3.749), p<.001. Further, findings revealed that older LGBT adults tend to engage in more risky or unhealthy behaviors when lonely than do heterosexual older adults (t=3.907, p<.001). Overall, the results indicate that while LGBT older adults may spend more time alone (t=7.350, p<.001), they are engaging in different types of activities, particularly involving technology to communicate with friends, suggesting compensation for their lack of in-person contact. By understanding how LGBT older adults cope with loneliness along with the risks and resources that have influences on their health disparities can be useful for developing interventions to improve the health and well-being in these communities.

